# Development of a multivalent adjuvanted inactivated vaccine against variant arthrotropic avian reoviruses

**DOI:** 10.3389/fvets.2023.1209597

**Published:** 2023-10-18

**Authors:** Lisanework E. Ayalew, Shelly Popowich, Betty Chow-Lockerbie, Hemlata Gautam, Iresha Subhasinghe, Khawaja Ashfaque Ahmed, Suresh K. Tikoo, Davor Ojkic, Susantha Gomis

**Affiliations:** ^1^Department of Veterinary Pathology, Western College of Veterinary Medicine (WCVM), University of Saskatchewan, Saskatoon, SK, Canada; ^2^Vaccinology and Immunotherapeutic Program, School of Public Health, University of Saskatchewan, Saskatoon, SK, Canada; ^3^Animal Health Laboratory, Laboratory Services Division, University of Guelph, Guelph, ON, Canada

**Keywords:** emerging ARVs, inactivated vaccine, multivalent, broiler breeders, broiler progenies

## Abstract

Variant avian reoviruses (ARVs) are economically important emerging pathogens of poultry, which mainly affect young broiler chickens and cause significant production losses. Currently, there are no effective commercial vaccines available for control and prevention of emerging variant ARVs. In this study, monovalent inactivated adjuvated (20% Emulsigen D) broiler breeder vaccines containing antigens from ARV genotype cluster (C) group -2, -4, -5, or -6, and a multivalent vaccine containing antigens from all the four indicated genotypic cluster groups were developed and evaluated for their efficacy in protecting broiler progenies against homologous or heterologous ARV challenge. The use of monovalent or multivalent inactivated vaccines in a prime-boost immunization strategy induced the production of ARV specific antibodies in broiler breeders. The maternal antibodies were effectively transferred to broiler progenies. Broiler progenies obtained from immunized breeders demonstrated milder clinical symptoms and reduced gross and histopathological lesions after homologous ARV challenge. More severe gross and histological lesions were observed in challenged progenies from unvaccinated broiler breeders. However, cross protection was not observed when either of the monovalent-vaccine groups were challenged with a heterologous virus. In addition, the progenies from the unvaccinated ARV challenged control or heterologous ARV challenged vaccinated groups had significantly reduced body weight gain (*p* < 0.01) than the unchallenged-control, challenged-multivalent, or homologous ARV-challenged monovalent vaccine groups. However, homologous ARV challenged progenies in the multivalent or monovalent vaccine groups had similar body weight gain as the control unchallenged group with significantly reduced viral load (*p* < 0.01) in the gastrocnemius tendon tissue. This study indicates that broad-spectrum protection of broiler progenies from variant ARV infections is feasible through the development of multivalent vaccines after proper characterization, selection and incorporation of multiple antigens based on circulating ARV genotypes in targeted regions.

## Introduction

Arthrotropic avian reoviruses (ARVs) are among the most economically significant pathogens of poultry, which are associated with unilateral or bilateral tenosynovitis/arthritis (1, 2). ARVs are non-enveloped segmented double-stranded RNA viruses with two icosahedrally concentric protein shells. The particle size ranges between 70 and 80 nm in diameter (3–5) and are resistant to heat, proteolytic enzymes, various disinfectants, and a wide spectrum of pH (6). ARV associated disease is most common in broiler chickens and mainly affects the weight bearing hock joint resulting in lameness, poor growth, poor production, and increased mortality (7–11). In addition, the ARV mediated damage to the gastrocnemius tendon tissue is mediated by interferon-⋎ producing CD8+ T cells and infection clearance is mainly mediated by humoral immunity (12, 13). In the past decade, despite the use of commercial vaccines, there has been a drastic increase in ARV associated disease outbreaks with emerging variants in the poultry industry across many geographic locations in Canada and USA, causing considerable economic losses and animal welfare issues (7–11).

We previously isolated several ARVs from broilers showing clinical signs of lameness and performed a comprehensive analysis of their phenotypic, genetic, and antigenic characteristics (7, 14). We demonstrated that the emerging virulent circulating strains were genetically diverse and evolutionarily distant from the vaccine and vaccine related field strains. Moreover, several reports (7–11, 15, 16) support the evidence that the emerging ARVs can break vaccine induced immunity resulting in the persistence of ARV associated disease in poultry flocks. However, despite their genetic heterogeneity, virulent ARV genotypes had similar pathotype features with semi-conserved determinants of virulence factors (13) which makes classification of isolates based on pathotype and association with genotypic classification very difficult (17). Because of the absence of commercial vaccines against the currently circulating emerging ARV variants (17, 18), the poultry industry in North America uses autogenous vaccines as alternatives to control and prevent the ARV associated disease (19, 20). The only commercial trivalent inactivated vaccine which is based on variant ARV serotypes (i.e., serotypes 1/4455, 2/4455, and 3) in North America is Avian Reovirus Vaccine^TM^. The product was recently developed by Ceva Biomune (18). The effectiveness of autogenous vaccines can be compounded by several factors including the co-circulation of multiple antigenic variants in the farm/region and lack of robust characterization of circulating variants and antigen inclusion criteria (18). Therefore, the objective of this study was to develop an adjuvated broad-spectrum multivalent inactivated ARV broiler breeder vaccine containing antigens from four genotyping groups, which were previously characterized at the full genome level (13) and evaluate the efficacy of maternal antibodies in protecting broiler progenies from disease after challenge with either of the four ARV genotypes individually.

## Materials and methods

### Viruses, cell lines, and media

Plaque purified prototype arthrotropic avian reoviruses (ARVs) representing genotype cluster group (C)- 2, 4, 5, and 6 (7, 13, 14) were used in this study. Leghorn male hepatoma (LMH) cell line (ATCC^®^) was used for virus propagation, virus neutralization and virus load determination. The LMH cells were grown in Dulbecco's Modified Eagle medium (DMEM)-12 (Life Technologies) supplemented with 20 mM HEPES, 2 mM L-glutamine, 50 mg/ml gentamicin and 10% fetal calf serum.

### Inactivated vaccine formulation

Prototype ARVs from genotyping cluster group (C)- 2, 4, 5 and 6, which were isolated in Saskatchewan, Canada and characterized for their genetic, antigenic, and phenotypic properties (7, 14), were used for vaccine formulation. Previously, LMH cells were infected at a multiplicity of infection (MOI) of 0.1 to prepare parent virus stock preparation. For inactivated vaccine preparation, LMH cells grown at 80% confluency on T-75 cell culture flasks (ThermoFisher Scientific) were infected with individual prototype viruses at an MOI of 5. The cells were harvested 48 h post infection, freeze-thawed 4 times, centrifuged at 3,000 rpm for 15 min. Next, the supernatants were collected, and virus was purified by density gradient ultra centrifugation method as described earlier (7). The identity of each virus was confirmed by polymerase chain reaction (PCR) and sequencing of the Sigma-C gene using specific primers as reported before (7). Each virus titer was quantified by TCID_50_ using LMH cells grown on 96 well tissue culture plates. Virus inactivation was performed by adding 0.2% formalin (v/v) to each virus (i.e., 1 × 10^8^ TCID_50_) and incubating at 37°C for 36 h. Viability of the virus was tested by inoculating LMH cells and monitoring the development of ARV specific cytopathic effect (CPE) over 7 days. The monovalent vaccines were prepared by separately mixing each inactivated virus (1 × 10^8^ TCID_50_) with 20% Emulsigen-D (MVP Laboratories, and Omaha, Nebraska). The multivalent (i.e., incorporating the four genotyping cluster groups: C2, C4, C5 and C6) inactivated vaccine was prepared by mixing antigens from each respective inactivated ARV genotype (2.5 × 10^7^ TCID_50_) with 20% Emulsigen-D. The final volume of each vaccine preparation was 0.5 ml/bird.

### Maintenance and vaccination of broiler breeders

ARV free day-old Ross broiler breeders were sourced from Aviagen Inc. (Huntsville, Alabama, USA). The breeders were confirmed for their negative status for ARV infection by enzyme linked immunosorbent assay (ELISA). The birds were then divided into six groups, 23 birds/group [i.e., cluster (C)-2, -4, -5, -6, multivalent, and unvaccinated negative control groups] and housed in the containment level-1 facility at the animal care unit (ACU) of the Western College of Veterinary Medicine (WCVM), University of Saskatchewan. Three of the birds were roosters and the rest were hens. The birds were reared as per Aviagen guidelines. The first dose of each vaccine [i.e., the 1^st^, 2^nd^, 3^rd^, 4^th^, and 5^th^ group were vaccinated with inactivated cluster (C)-2, -4, -5, -6, and multivalent vaccine, respectively and the 6^th^ group was kept as unvaccinated control group] was administered at 14 weeks of age to both hens and roosters via the intramuscular route on the breast muscle. Each group received a booster dose of the same vaccine preparation via the same route of administration at 17 weeks of age. The flock started production at 25 weeks of age, and eggs were collected between 28 and 34 weeks of age to set and hatch for broiler progeny. The average fertility rate was 98%.

### Enzyme linked immunosorbent assay

Before vaccination of broiler breeders, serum was collected and tested for the presence of anti-reovirus antibodies by avian reovirus ELISA (IDEXX Laboratories, USA). A cut-off value of 396 (S/P ratio > 0.20) and above was considered positive as indicated by the manufacturer. Twenty-one days post prime vaccination, and 15 days post booster vaccination, serum samples were collected from all the groups and the level of seroconversion was evaluated by the IDEXX ELISA.

### Virus neutralization test

Serum samples collected from broiler progenies (*n* = 5/group) were heat inactivated at 56°C for 30 min. The virus neutralization activity of the sera collected from the respective vaccine or negative control groups were tested against wild-type homologous ARV. Briefly, the inactivated sera were serially diluted (2-fold) in a 96 well plate and incubated with 200 TCID_50_ of homologous ARV and incubated for 1 h at 37°C. The virus-sera mixture was then transferred to freshly grown LMH cells (5 × 10^4^ cell/well) in 96 well plates. Virus infected and uninfected LMH cells were used as negative controls. Finally, the plates were transferred to a humidified 37°C incubator and observed for the development of cytopathic effect (CPE) every day for 7 days.

### Broiler progeny challenge with homologous or heterologous ARV

Broiler progenies (*n* = 30) obtained from each group (i.e., vaccinated or unvaccinated) were tagged and housed in separate rooms in a level-2 facility in the WCVM animal care unit. At 7 days of age, each group was challenged with either a heterologous or homologous ARV virus with a total of 1 x 10^5^ TCID_50_ virus particles via the right footpad route. Similar challenge dose was used for each virus because these viruses do have similar pathotype features (7, 13). A virus was designated as heterologous based on negative results on virus neutralization assay using antibodies produced against ARV from another genotypic cluster group (7, 13). The birds were monitored for the development of clinical signs every day for 30 days. From each group, three birds were euthanized by cervical dislocation at different time points post infection and, tendon and spleen samples were collected for virus load determination. Histopathology was performed on the tendon tissues. The body weight of the birds in each group was measured and recorded at 3-, 6-, 16-, and 30-day post infection. Gross footpad lesion scoring was performed at 3, 6 and 30-day post infection. The relative mean body weight of the birds in each group was calculated and compared with the mean body weight of the control group.

### Virus load quantification by tissue culture infectious dose 50

Equal amount of tendon tissue or spleen collected at day-3, -6, -16, and -30 post infection from vaccinated/challenged or unvaccinated/challenged grouped were collected in 1.5 ml RINO™ screw-cap microcentrifuge tube with magnetic beads (Next Advance, Inc., NY, United States) and homogenized using a bullet blender storm-24 (Next Advance, Inc., NY, United States) for 10 min at maximum speed. The homogenized material was used to determine virus load in each sample by TCID50 as previously described (13). The differences in virus load between the vaccinated and the control groups were analyzed by a one-way ANOVA analysis.

### Histologic examination

Sections of gastrocnemius tendon tissues collected from birds from each group were fixed in 10% neutral buffered formalin and, processed for sectioning and Hematoxylin-Eosin staining as described earlier (7, 13) and the slides were analyzed using a light microscope for histologic lesion scoring.

### Statistical analysis

Data analysis was performed using GraphPad Prism 9 (Graph Pad Inc., San Diego, CA). The mean body weight and mean virus load in tendon tissues were compared between different groups by one-way analysis of variance (ANOVA) with statistical significance level of *p* < 0.05.

### Ethics statement

The animal study protocol was reviewed and approved by the University of Saskatchewan's Animal Care Committee (UACC) Animal Research Ethics Board (AREB; Certificate of approval #: 20160010) and was conducted according to the Canadian Council on Animal Care (CCAC) guidelines.

## Results

### Confirmation of ARV genotypes by Sigma-C gene sequencing and virus inactivation

The Sigma-C gene of plaque purified prototype ARV from each genotypic group was PCR amplified and sequenced. Nucleotide BLAST of the sequences confirmed the identity of the ARVs [S1 genomic segment sequence GenBank Acc #s for genotyping cluster C2 = MN879660, C4 = MN879600, C5 = MN879610, and C6 = MN879700 (14)] used for vaccine formulation. While inoculation of LMH cells with live ARVs produced virus specific CPE within 24 h., inoculation of the LMH cells with respective killed ARVs did not produce any CPE for the 7-day observation period which confirmed successful virus inactivation.

### Vaccine induced seroconversion in broiler breeders

**Figure 1:**
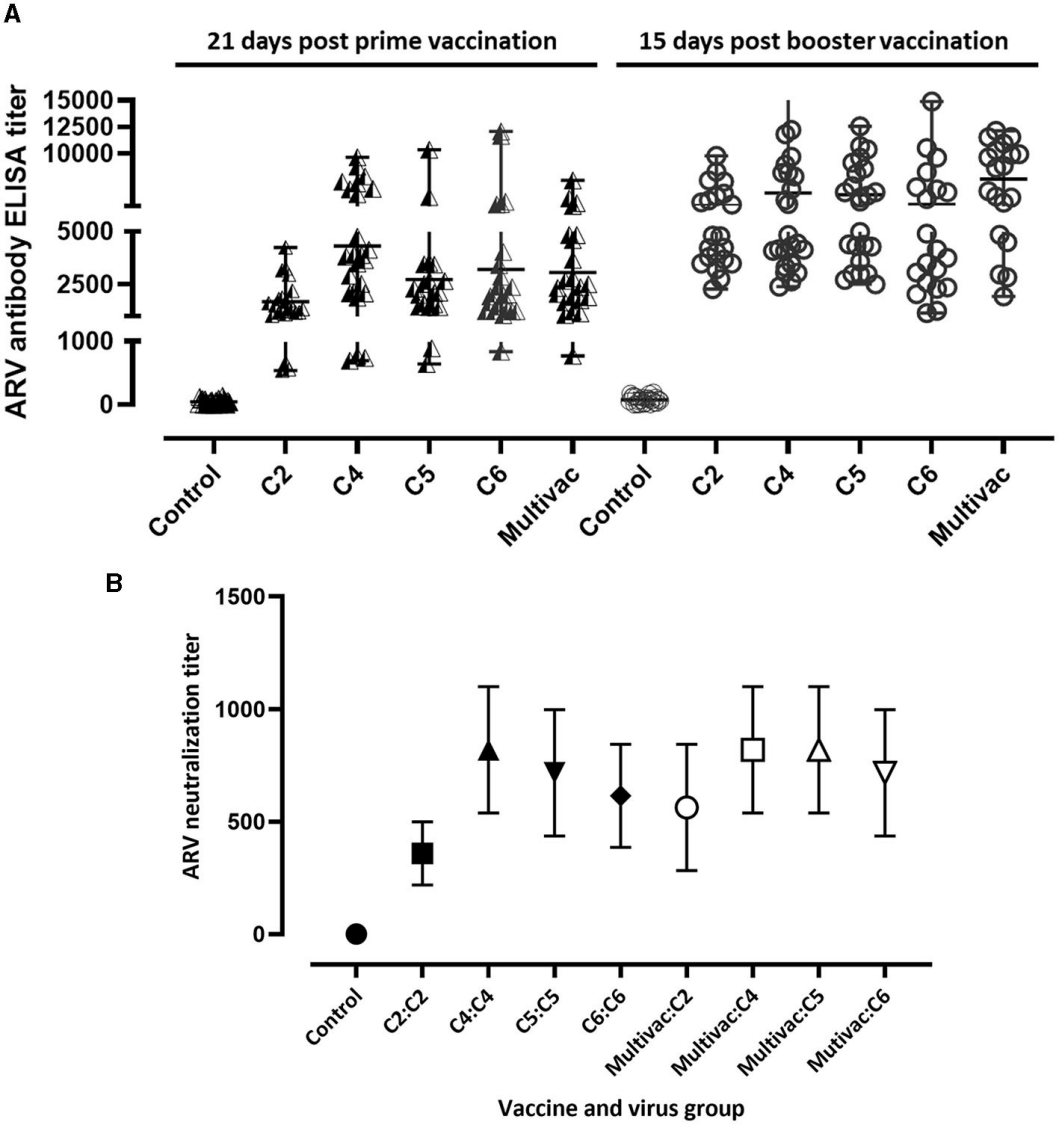
Serology. **(A)** ARV ELISA titer in broiler breeders 21 days prime vaccination and 15 days post booster vaccination; C2, C4, C5, or C6 represent inactivated vaccine prepared from ARV genotyping cluster group (C)-2, 4, 5, or 6 respectively. Multivac: Inactivated multivalent vaccine containing antigens from all the indicated ARV antigenic groups. **(B)** ARV neutralizing maternal antibody titer in day-old broiler progenies obtained from monovalent inactivated ARV vaccine [genotyping cluster group (C)-2, 4, 5, or 6] tested against homologous virus, multivalent inactivated ARV vaccine (Multivac) tested against either of the four indicated ARV genotypes or unvaccinated-control broiler breeders.

To study the immunogenicity of the inactivated ARV vaccine antigens, we immunized broiler breeders with adjuvated monovalent ARV vaccines or multivalent inactivated ARV vaccine [i.e., containing antigens from four cluster groups: Cluster (C)-2, C4, C5, and C6] and progenies obtained from the vaccinated broiler breeders were challenged with homologous or heterologous ARV genotypes. The degree of seroconversion in experimental broiler breeder groups was measured 21 days post prime vaccination. Except for the unvaccinated control group, the monovalent and multivalent groups seroconverted (Figure 1A). Broiler breeders vaccinated with monovalent C2, C4, C5, C6, or multivalent (i.e., containing antigens from all the four genotypic groups) inactivated ARV vaccines had mean titers of 1678.78 (±928.52), 4308.93 (±2614.69), 2731.37 (±2194.45), 3205.96 (±3058.93) and 3063.36 (±1820.25), respectively, 21 days post prime vaccination (Figure 1A). The mean titers for C2, C4, C5, C6, and the multivalent vaccine groups were 5034.86 (±1912.44), 6244.46 (±3740.06), 6078.92 (±2852.62), 5143.45 (±3533.80), and 7572.12 (±3275.06), respectively, 15 days post booster vaccination (Figure 1A).

### Virus neutralizing maternal antibody titer in broiler progenies

Serum samples were collected from day old broiler progenies which were obtained from unvaccinated control and vaccinated broiler breeders to measure the level of neutralizing maternal antibody titers. The mean homologous ARV maternal neutralization antibody titers in the monovalent vaccine groups; C2, C4, C5 and C6 were 358.4 (±140.22), 819.2 (±280.43), 716.8 (±280.43), and 614.4 (±228.97), respectively (Figure 1B). Cross maternal antibody neutralization against heterologous ARVs was not observed. The mean maternal neutralization antibody titers against ARVs in genotyping cluster group (C) 2, 4, 5, and 6 in broiler progenies obtained from the multivalent broiler breeder vaccine group were 563.2 (±280.43), 819.2 (±280.43), 819.2 (±280.43), and 716.8 (280.43), respectively (Figure 1B).

### Clinical signs and gross pathological lesions in ARV challenged broiler progenies

**Figure 2:**
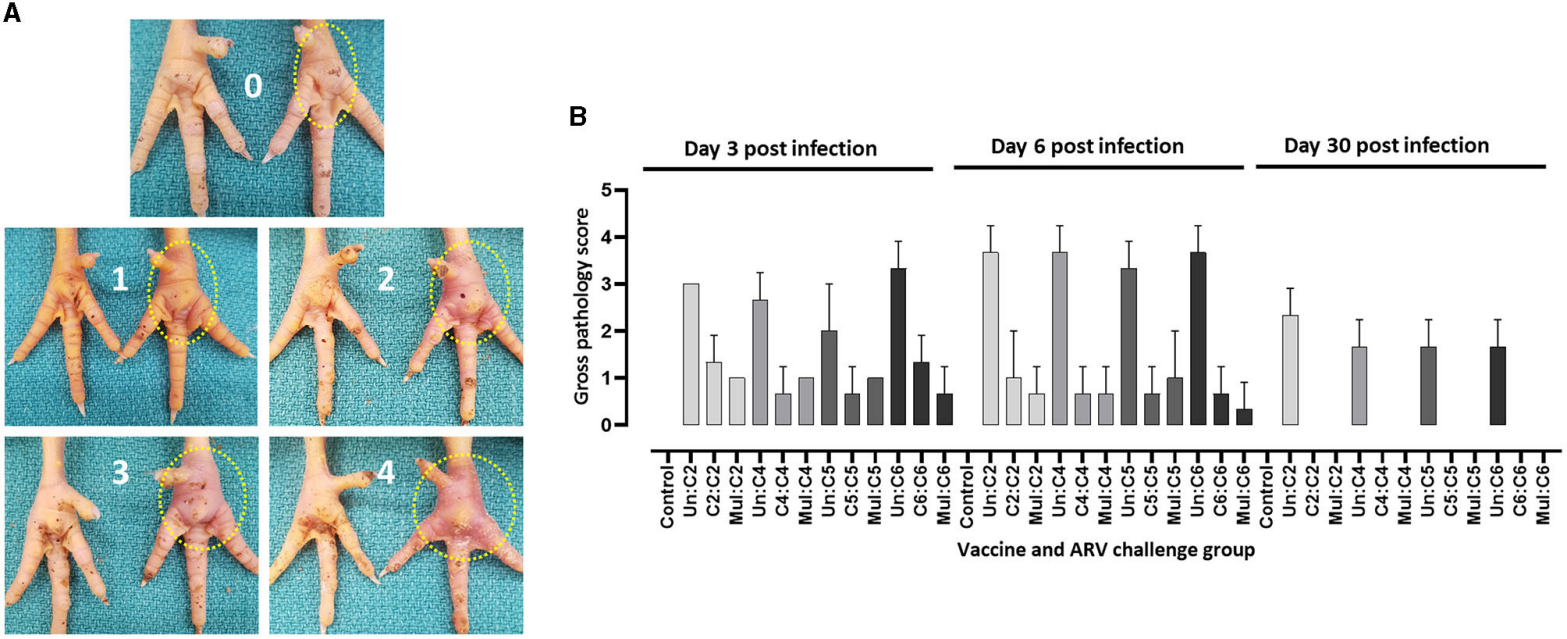
**(A)** Scoring of macroscopic lesions on the right footpad of birds infected with ARV. **(B)** Macroscopic scores of footpad lesions in broiler progenies from vaccinated broiler breeders with a monovalent inactivated ARV vaccine [i.e., antigens representing genotyping cluster group (C)-2, 4, 5, or 6], a multivalent vaccine (Mul) or unvaccinated (Un) controls at days 3, 6, and 30 post challenge with a homologous ARV.

The most prominent signs observed in broiler progenies from unvaccinated control breeders were depression and significant swelling of the footpad. These birds were lame and struggled to walk or tried to support their weights using their wings to reach for food or water. Most of the birds did not respond well to visual, tactile, and acoustic stimuli. Similar clinical signs were observed in broiler progenies obtained from broiler breeders that received monovalent inactivated ARV vaccine and challenged with a heterologous ARV. On the contrary, broiler progenies obtained from broiler breeders vaccinated with the monovalent vaccine and challenged with a homologous ARV or broiler progenies obtained from broiler breeders vaccinated with a multivalent vaccine and challenged with either C2, C4, C5 or C6 ARVs were very active with few having minor footpad inflammation and responded well to different stimuli. Gross lesion scoring of the ARV (i.e., either of the four genotyping cluster groups) infected footpad of progenies obtained from vaccinated and unvaccinated boiler breeders was made as follows: 0 = no inflammation; 1 = slight inflammation with reddening of the skin around the footpad; 2 = Inflammation with reddening of the skin and slight swelling of the footpad; 3 = Inflammation with reddening of the skin and moderate swelling of the footpad; 4 = Inflammation with reddening of the skin and prominent swelling of the footpad which may extend up to the hock joint (Figure 2A). The footpad gross lesions clinically scored at day 3-, 6-, and 30 post-infection. A prominent inflammation and swelling of the footpad were observed in all unvaccinated and challenged groups with either of the four ARV genotypes at day 3 post infection. The severity of the inflammation increased at day 6 post infection and reduced significantly at day 30 post infection (Figure 2B). The maximum gross pathology score recorded in the monovalent (after homologous challenge) or multivalent vaccine group was inflammation with reddening of the skin and slight swelling of the footpad. Gross pathology was not observed after day 6 post infection in all the vaccinated groups challenged with homologous virus. No lesions were observed in boiler progenies in the unchallenged control group (Figure 2B).

### Histologic lesions in ARV challenged broiler progenies

**Figure 3:**
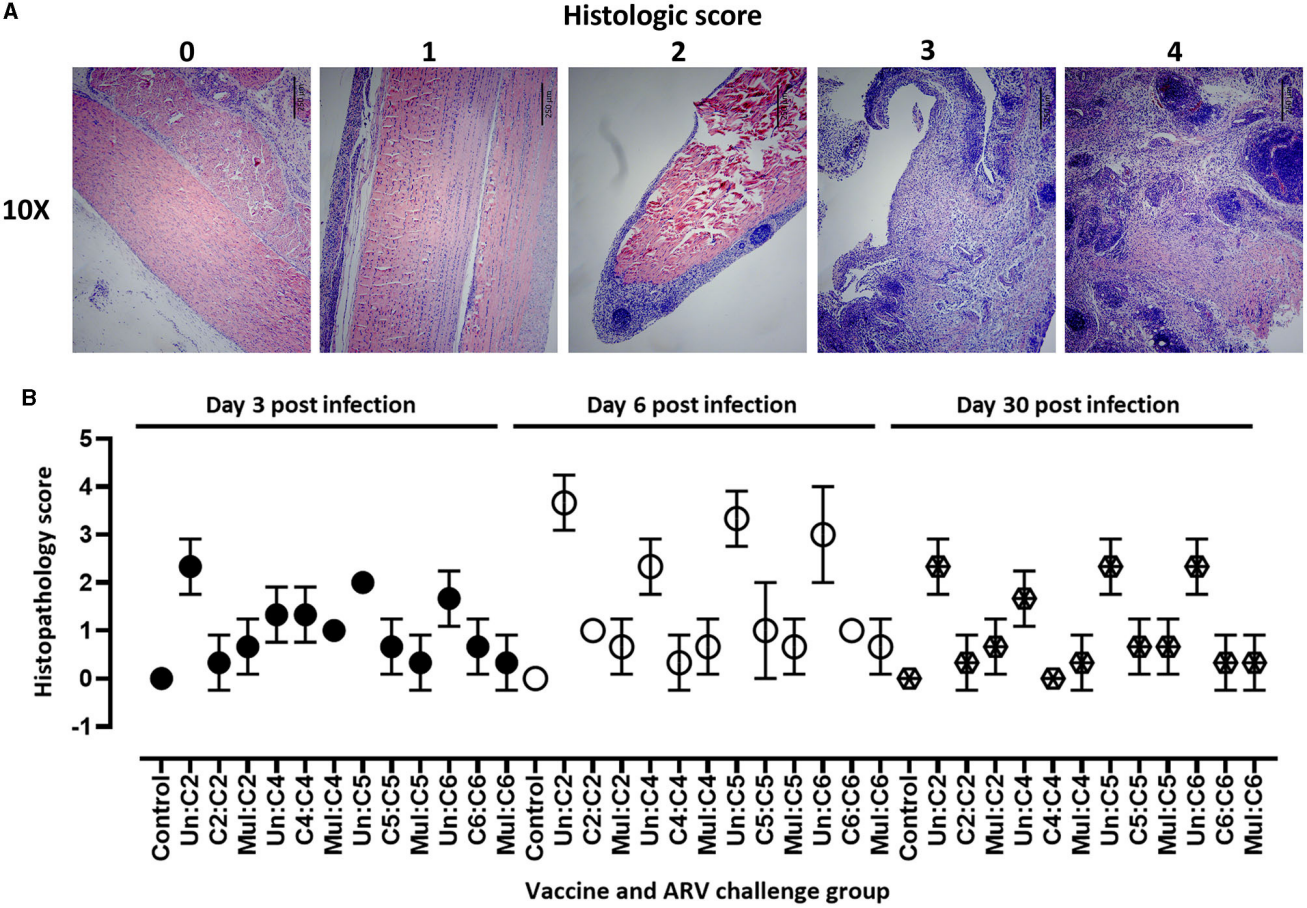
**(A)** Scoring of histopathologic lesions of tendon tissue of birds infected with ARV. 0 = normal; 1 = mild tendinitis; 2 = moderate tendinitis; 3 = moderate to severe tendinitis; and 4 = severe tendinitis. Scale bars: 250 μm. **(B)** Histopathologic scores of tendon tissues in broiler progenies from vaccinated broiler breeders with a monovalent inactivated ARV vaccine [i.e., antigens representing genotyping cluster group (C)-2, 4, 5, or 6], a multivalent vaccine (Mul) or unvaccinated (Un) controls at days 3, 6, and 30 post-challenge with homologous ARVs.

For detailed analysis of the pathology, histopathology was performed on tendon tissue samples collected from broiler progenies from vaccinated or unvaccinated breeders and challenged with homologous and heterologous ARV. The histopathology lesions were scored as follows: 0 = normal; 1 = mild tendinitis; 2 = moderate tendinitis; 3 = moderate to severe tendinitis; and 4 = severe tendinitis (Figure 3A). Histopathology of tendon tissue samples from all experimental groups were graded on days 3, 6, and 30 post-infection. The most severe histologic lesions were observed on day 6 post infection as compared to days 3 or 30 post-infection in unvaccinated groups challenged with ARV from either of the four cluster groups (Figure 3B) and in monovalent vaccine groups challenged with a heterologous virus (data not shown). The histological lesions observed in monovalent or multivalent vaccine groups challenged with homologous ARV were significantly lower than the control groups at all the analyzed time points post infection. No histological lesions were observed in the unchallenged negative control groups (Figure 3B).

### Body weight gain in broiler progenies challenged with homologous or heterologous ARV

**Figure 4:**
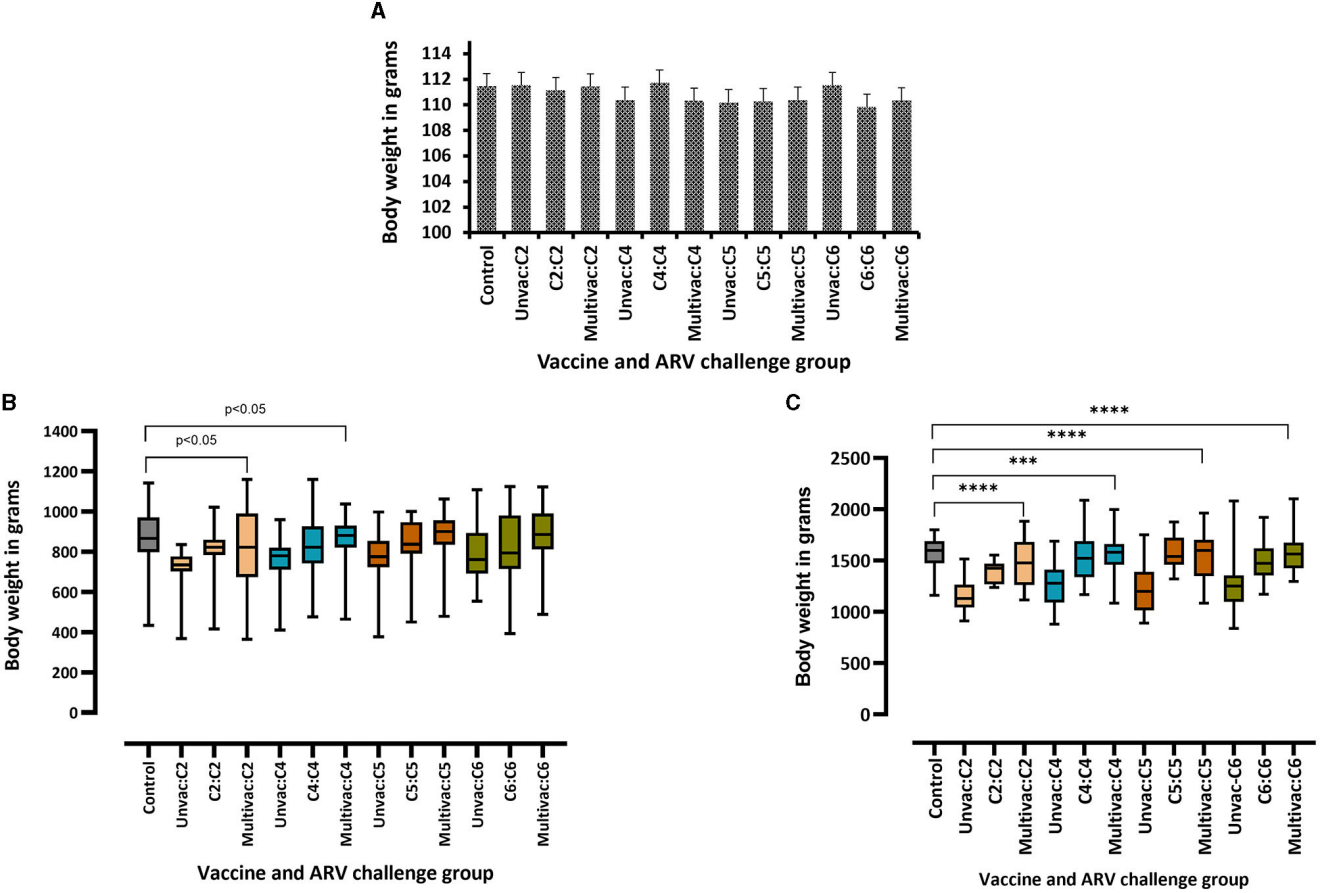
Body weights of broiler progenies from vaccinated broiler breeders with a monovalent inactivated ARV vaccine [i.e., antigens representing genotyping cluster group (C)-2, 4, 5, or 6], a multivalent vaccine (Multivac) or unvaccinated (Unvac) controls measured on the day of challenge **(A)**, and days 16 **(B)**, and 30 **(C)** post-challenge with homologous ARVs. ***p = 0.0001, ****p < 0.0001.

The body weight of the broiler progenies in the different experimental groups was measured on days 3-, 6-, 16-, and 30 post ARV infection as a measure of vaccine protection. No significant difference in body weight was observed at the day of challenge between the different groups (Figure 4A). There was also no significant difference in body weight on days 3 and 6 post challenge between challenged broiler progenies obtained from vaccinated breeders, unvaccinated breeders or unchallenged control groups (data not shown). However, the body weight gain of unvaccinated challenged groups started to reduce at day 16 post infection as compared to the unvaccinated/uninfected control group. The monovalent vaccine group or multivalent vaccine group challenged with a homologous ARV from genotypic cluster group-2 (C2) and -4 (C4) had significant reduction (*p* < 0.05) in body weight at day 16 (Figure 4B). Again, at day 30 post infection, the body weight gain was significantly reduced (*p* < 0.0001) in all unvaccinated-challenged groups than the vaccinated-homologous ARV challenged groups or the unchallenged control groups (Figure 4C). Taking body weight gain as a measure of protection, no cross-protection was conferred when broiler progenies were challenged with a heterologous ARV.

### ARV levels in the tendon and spleen tissues of challenged broiler progenies

**Figure 5:**
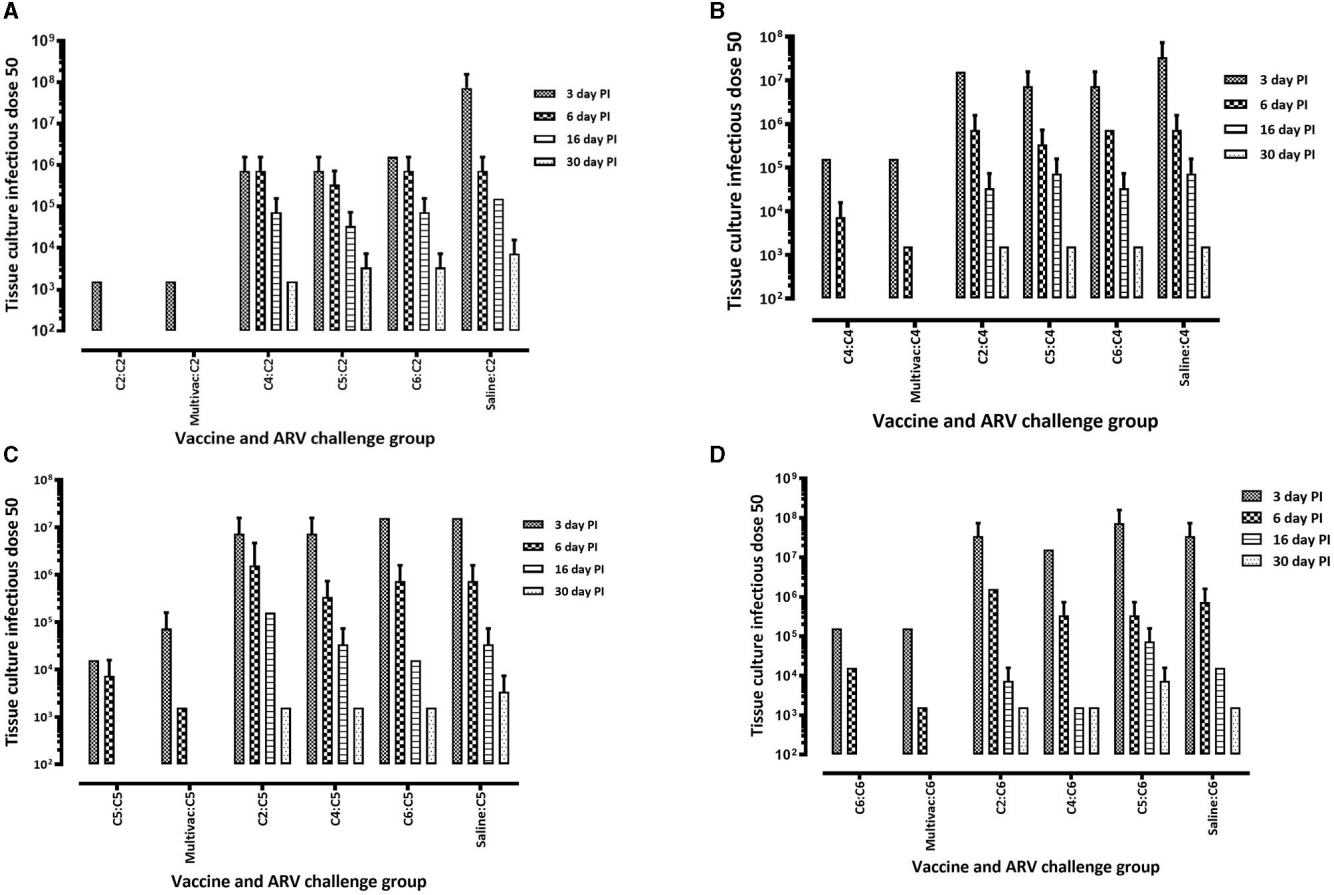
Virus load in the tendon tissue of broiler progenies from vaccinated breeder parents with monovalent [i.e., antigens representing genotyping cluster group (C)-2, 4, 5, or 6] or a multivalent inactivated vaccine (Multivac). Each vaccine group was challenged with **(A)** reovirus cluster group-2 (C2); **(B)** reovirus cluster group-4 (C4); **(C)** reovirus cluster group-5 (C5); **(D)** reovirus cluster group-6 (C6).

The protective efficacy of the adjuvated monovalent or multivalent inactivated ARV vaccines were further evaluated by determining the vaccines ability to suppress the replication of ARV locally in infected tendon tissues, and systemically in the spleen of broiler progenies. This was examined by measuring ARV loads in the tendon and spleen tissues at days 3, 6, 16, and 30 post-homologous or heterologous ARV challenge. Broiler progenies obtained from broiler breeder groups vaccinated with monovalent or multivalent vaccine and challenged with a homologous ARV genotype cluster (C)-2 (Figure 5A), C-4 (Figure 5B), C-5 (Figure 5C) or C-6 (Figure 5D) had significantly lower virus load in the tendon tissues than the virus load in the tendon tissues of broiler progenies, which were challenged with a heterologous ARV genotype or unvaccinated-challenged control groups. No virus was detected in the unchallenged control group.

**Figure 6:**
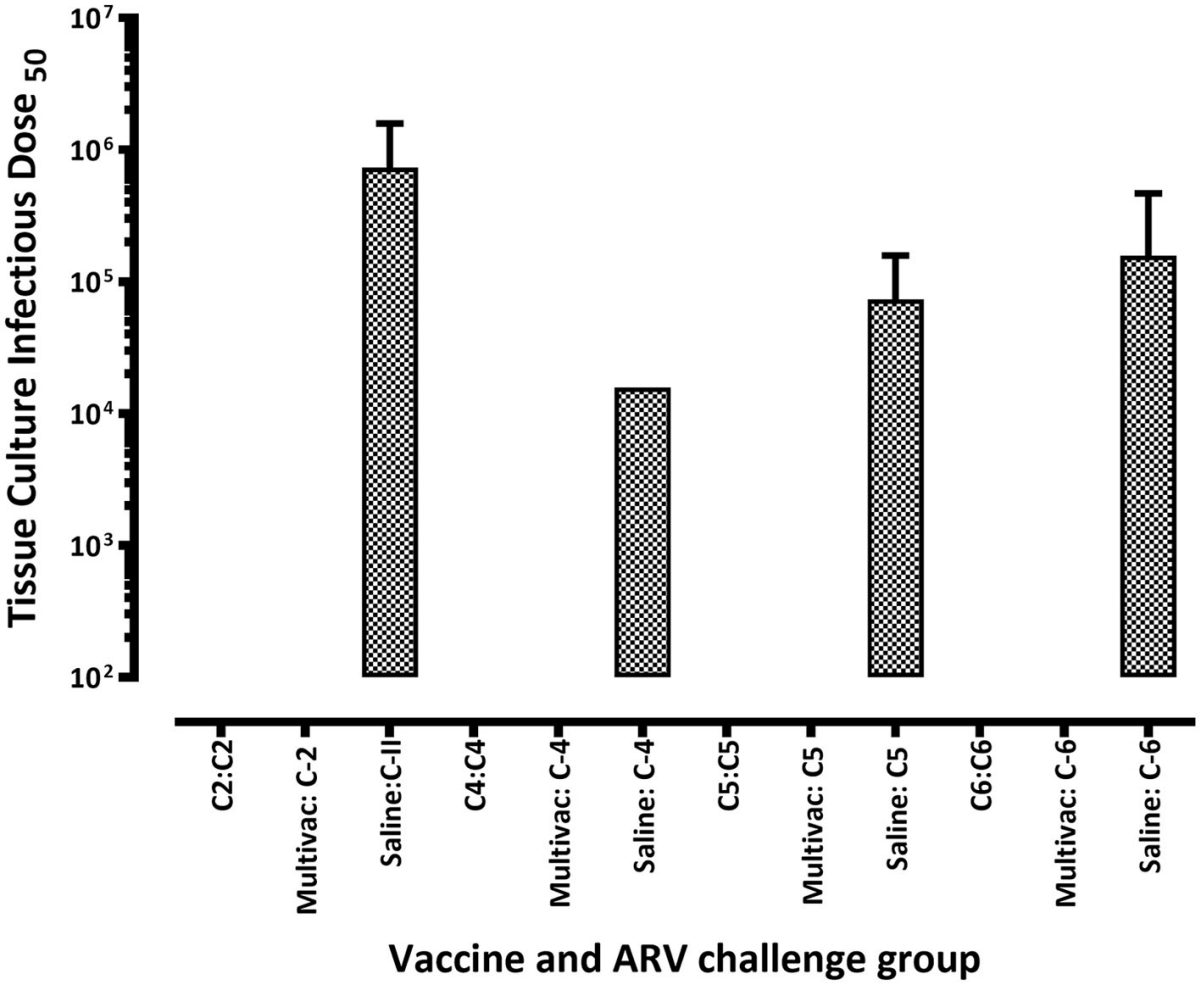
Virus load in the spleen of broiler progenies from vaccinated breeder parents with monovalent [i.e., antigens representing genotyping cluster group (C)-2, 4, 5, or 6)] or a multivalent inactivated vaccine (Multivac) post homologous virus challenge.

In the homologous ARV challenged broiler progenies, virus was not detected on days 16 and 30 post infection (Figures 5A–D). However, ARV was detectable on day 30 post infection in heterologous ARV challenged broiler progenies or in unvaccinated-challenged control groups (Figures 5A–D). Interestingly, virus was not detected in the spleen tissues of all broiler progeny vaccine groups at all time points analyzed after challenge with a homologous ARV genotype (Figure 6). Nonetheless, virus was detected in the ARV challenged saline control groups at 3 days post-infection but not at day 6-, 16-, or 30 post-infection (Figure 6).

## Discussion

Avian reoviruses (ARVs) are etiological agents for an economically important poultry disease (6, 17, 19). Arthrotropic ARVs cause tenosynovitis/arthritis syndrome, characterized by unilateral or bilateral swelling of the hock joint resulting in lameness, production loss and animal welfare concerns (8–10, 21). The most widely used historical commercial vaccines against ARV induced tenosynovitis/arthritis include the live S1133 vaccine, which was developed through serial passages in embryonating chicken eggs and in chicken embryo fibroblast (CEF) cells (17, 22) and live and inactivated vaccines based on strains 2,408, 1,733 including naturally non-virulent immunogenic strain 2177 (1, 17, 19). However, these vaccines fail to protect broilers against disease produced by emerging ARV variants (7–11, 23). Recent studies on ARVs have mainly focused on the genetic characterization of clinical isolates. However, there are very limited studies available on vaccine development against the emerging ARV variants. Currently, no effective commercial vaccine is available against the circulating variant ARVs in the North American broiler chicken industry (18, 19). The alternative method which is being employed to control ARV infection in the region is by using autogenous vaccines (17–19). However, autogenous vaccines may have limited potential as dedicated control measures against ARVs. Therefore, the objective of this study was to develop and test the effectiveness of adjuvated multivalent inactivated broiler breeder vaccines for the broad-spectrum protection of broiler progenies against infection by four different antigenic variants of ARV.

Our adjuvated monovalent or multivalent inactivated ARV vaccines used in a prime-boost strategy induced the production of ARV antibody responses. The maternal antibodies transferred to broiler progenies neutralized homologous ARVs but not heterologous ARVs *in-vitro* on cell culture. This is in line with our previous study which demonstrated a significant antigenic variability between the viruses grouped into different genotyping cluster groups by structure-based analysis of predicted epitopes on the σ-C protein (7). Meanger et al. (24) also reported that antibodies produced against a heterologous ARV were type specific and not cross-neutralizing.

Disease outbreaks associated with variant ARVs occur in the field despite vaccination of chickens with commercial or autogenous vaccines (8–11) suggesting the absence of cross neutralization of ARVs by antibodies induced by heterologous antigens. The level of neutralizing maternal antibody transfer to broiler progenies could be increased by administering additional booster doses to the broiler breeders.

Similarly, our *in-vivo* challenge studies in broiler progenies obtained from broiler breeders immunized using either homologous monovalent vaccine antigens or multivalent vaccine had milder clinical symptoms and, significantly reduced gross and histopathological lesions than the unvaccinated- ARV challenged control groups or immunized groups with monovalent vaccine antigens with a heterologous virus challenge. This data corroborates the *in-vitro* virus neutralization assay suggesting that the functional antigenic epitopes of the different ARV groups are significantly variable and optimal cross-protection may not be achieved by a heterologous vaccine.

One of the impacts associated with arthrotropic ARV infections in broiler chickens is decreased body weight gain (9, 13, 18, 19), increased feed conversion ratios and condemnations in chicken processing plants (17–19). Hence, body weight gain was used as one of the measures of vaccine protection in our study. As expected, the challenged-unvaccinated control groups and immunized groups with monovalent antigens and challenged with a heterologous virus had significantly lower body weight gain than the unchallenged control group or the multivalent vaccine group. This indicates that the most viable way to effectively control ARV associated tenosynovitis/arthritis in broiler chickens and prevent economic losses in the poultry industry is through the careful characterization of variant ARVs in the specific region and the development of broad-spectrum vaccine by including all the appropriate antigens in the vaccine formulation from representative variant ARVs.

In the monovalent antigen immunized-homologous ARV challenged groups or ARV challenged multivalent antigen immunized groups, virus was cleared from the tendon tissues after day 6-post infection further confirming the effectiveness of the neutralizing maternal antibodies transferred to broiler progenies. On the contrary, virus was detected in the tendon tissues of the unvaccinated-challenged control groups and the monovalent antigen immunized-heterologous ARV challenged groups through out the trial period. Previous studies suggest that replication of arthrotropic ARVs can persist in the hock joint of susceptible chickens for a long period (13, 25). Besides, arthrotropic ARVs could infect mononuclear phagocytes and spread systemically and replicate in internal organs like the spleen (13, 26). Our study indicates that the production of neutralizing antibodies against a homologous ARV can restrict the replication of the virus locally in the gastrocnemius tendon tissue and prevent the spread of the virus into other parts of the body.

One of the limitations of this study is that only one dose concentration and one adjuvant type were tested in a prime-boost strategy. We were unable to test different dose concentrations and different adjuvants in this project since the experiment would involve hundreds of other birds which would be costly and couldn't also be accommodated in our CL-2 animal facility at the time. The other limitation of this study is that for effective field application, priming with live attenuated vaccine followed by booster with inactivated vaccine would be more feasible as it would potentially induce an increased level of neutralizing antibody for a prolonged period in vaccinated broiler breeders. Since we are still in the process of attenuating prototype variant ARVs from each of the four genotypic cluster groups, we couldn't evaluate the effectiveness of a live prime-inactivated boost vaccination strategy.

In conclusion, the multivalent inactivated breeder vaccine was able to induce adequate antibody titers and the maternal antibodies transferred to broiler progenies conferred protection against challenge with any one of the four ARVs from the four genotypic cluster groups (C-2, C-4, C5, and C6). Overall, the data strongly supports the feasibility of developing a multivalent breeder vaccine which can provide broad-spectrum protection against tenosynovitis/arthritis in broiler chickens produced by infection with virulent variant ARVs.

## Data availability statement

The original contributions presented in the study are included in the article/supplementary material, further inquiries can be directed to the corresponding authors.

## Ethics statement

The animal study was approved by the University of Saskatchewan's Animal Care Committee (UACC) Animal Research Ethics Board (AREB; Certificate of approval #: 20160010). The study was conducted in accordance with the local legislation and institutional requirements.

## Author contributions

LA, SG, KA, ST, and DO conceptualized and designed the study. LA, SP, B-CL, HG, and IS performed the animal experiments and conducted sample collection. LA, B-CL, SP, and HG performed all the *in vitro* experiments. LA, SP, and HG performed the data analysis. LA wrote the first draft of the manuscript. All authors read and edited the manuscript and approved the final version for submission for publication.
